# A Case Report of Inferior Rectus Abscess

**DOI:** 10.21980/J8J35G

**Published:** 2025-04-30

**Authors:** Luke Chi, Adam Sauer, Danielle Matonis

**Affiliations:** * University of California, Irvine, Irvine, CA; ^ University of California, Irvine Medical Center, Department of Emergency Medicine, Orange, CA

## Abstract

Soft tissue infections, including abscesses, are frequently encountered in the emergency department. However, abscesses of the inferior rectus muscle are rare and may present unique diagnostic and therapeutic challenges due to the scarcity of documented cases. This case report highlights the treatment of a 47-year-old male presenting with an abscess in the left inferior rectus muscle due to an acute Methicillin-resistant Staphylococcus aureus infection. Imaging revealed a distinct fluid collection compressing the left globe, causing restricted ocular motility and other symptoms. Despite prior intravenous antibiotics, the abscess persisted. While intravenous antibiotics are an important component of treatment, this case highlights the potential insufficiency of this approach alone, emphasizing a need for surgical intervention such as orbitotomy for drainage. This report contributes to the limited literature on inferior rectus muscle abscesses and underscores the need for further research and clinical attention to optimize patient outcomes.

**Topics:**

Abscess, soft tissue infection, extraocular muscles, pyomyositis, Methicillin-resistant Staphylococcus aureus, proptosis, diplopia, vision loss.

**Figure:**
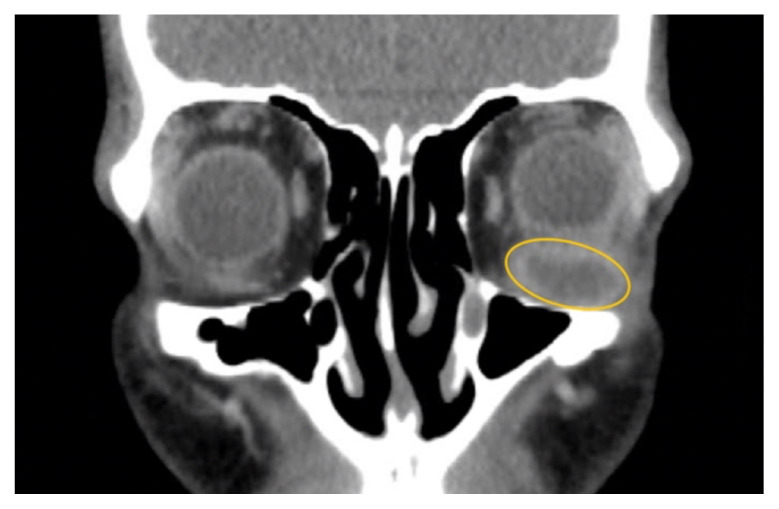


**Figure:**
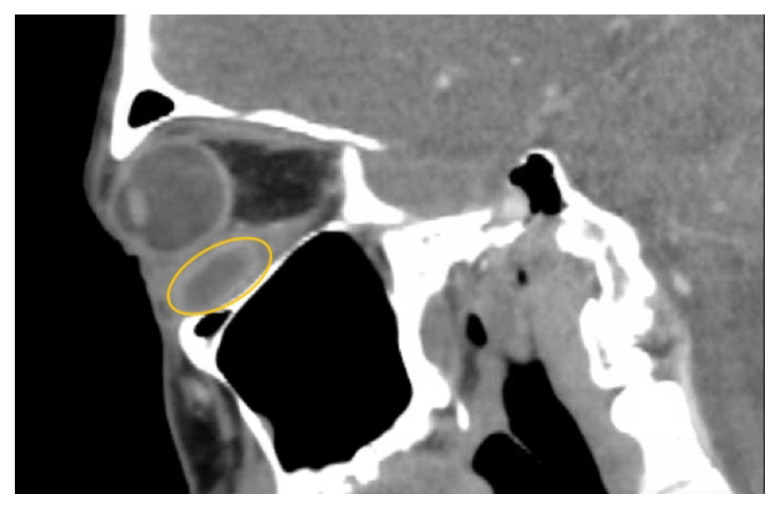


**Figure:**
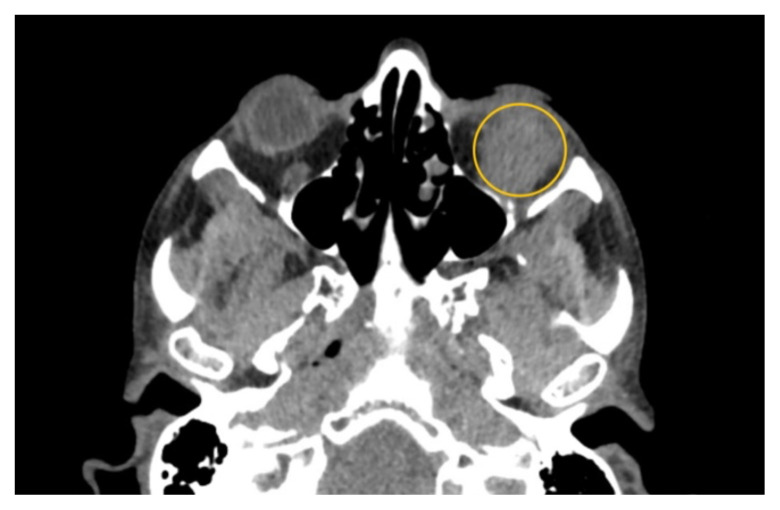


## Brief introduction

Inferior rectus muscle abscess is a rare condition with limited documentation in the medical literature. However, despite its rarity, early recognition and appropriate interventions are crucial for a full recovery.^1^ In one of the few documented case reports, permanent vision loss and impaired eye movement due to central retinal occlusion secondary to extraocular abscess formation have been documented.^2^ These instances underscore the importance of prompt identification and further documentation of similar cases.

This case report aims to provide a comprehensive overview of the clinical course and interventions for a male patient presenting with an abscess due to an acute Methicillin-resistant Staphylococcus aureus (MRSA) infection in the left inferior rectus muscle. Through this report, we aim to contribute to the limited body of literature on this rare condition and emphasize the importance of early diagnosis and appropriate management strategies.

## Presenting concerns and clinical findings

A 47-year-old male with a history of polysubstance use presented with ten days of left ocular pain and swelling as well as double vision. He recently left another hospital against medical advice, where he was diagnosed with an inferior orbital abscess and treated with intravenous (IV) antibiotics. Surgical intervention was recommended; however, the patient left due to extended wait times and later presented to our institution for a second opinion. He was afebrile with stable vital signs. On physical examination, the patient demonstrated intact visual acuity in both eyes, proptosis of the left eye, mild left periorbital swelling, restricted ocular motility in all directions (particularly with infraduction in the left eye), and binocular diplopia.

## Significant findings

Non-contrast computed tomography (CT) imaging of the head in coronal, sagittal, and axial planes revealed a distinct 1.7 × 2.2 × 1.4 cm peripherally enhancing fluid collection within the left inferior orbit, involving the inferior rectus (yellow circle). This lesion resulted in restricted extraocular motility due to structural compression of the left globe. Laboratory results showed a mildly elevated white blood cell count of 11.5/mm^3^ and otherwise normal results including C-reactive protein (CRP) and erythrocyte sedimentation rate (ESR).

## Patient course

The patient was started on IV antibiotics, ophthalmology was consulted, and he was admitted to the hospital. Despite completing one week of IV antibiotics between admission at the previous hospital and our institution, there was no appreciable change in the size of the abscess. Blood cultures were negative, and an echocardiogram did not reveal any findings suggestive of endocarditis. Ophthalmology planned for a left orbitotomy, incision and drainage, and surgical exploration; however, the patient ultimately declined this intervention and chose to leave the hospital against medical advice. During the inpatient course, the infectious disease service recommended IV vancomycin during hospitalization, followed by a four-week course of oral doxycycline upon discharge. The patient was strongly encouraged to continue the antibiotic regimen and follow up with ophthalmology as an outpatient for the surgical procedure. Unfortunately, the patient did not return to our healthcare system, and his long-term outcomes remain unknown.

## Discussion

Pyomyositis is an acute bacterial infection of the skeletal muscles that commonly manifests as abscesses and swollen regions.^3^,^4^ Abscesses caused by pyomyositis most frequently involve the muscles of the thigh and trunk.^5^,^6^ However, in rare instances, patients may present with pyomyositis involving the extraocular muscles.^5^,^7^ Recent literature has reported that muscles damaged by vigorous exercise and/or HIV infection are susceptible to pyomyositis; however, the precise mode of infection involving the extraocular muscles is not well understood.^8^,^9^ Certain hypotheses suggest that bacteria may access the extraocular muscles via the sinuses, adjacent anatomical regions, or via hematogenous spread.^8^,^9^ The most common clinical manifestations of pyomyositis involving the extraocular muscles include swelling, proptosis, pain, intermittent visual blurring, and restricted ocular motility which can vary depending on the location of the abscess.^2^ In severe cases, these abscesses may be situated near vital arteries and can lead to excessive swelling and vessel occlusion.^1^

When diagnosing abscesses and other soft tissue infections, the white blood cell count and inflammatory markers such as the Erythrocyte Sedimentation Rate (ESR) and C-Reactive Protein (CRP) are generally considered to be nonspecific. Multiple imaging modalities are available to identify and characterize these types of infections. Magnetic resonance imaging (MRI) is considered the most effective modality in delineating areas of fluid collection and areas of necrosis.^3^ Computer tomography is an appropriate alternative, especially if contrast can be utilized to better evaluate the soft tissue.^3^ When there is concern for necrotizing infection, X-rays and CT scans are considered more sensitive and specific for visualization of gas in the soft tissues, while there is disagreement among experts regarding the sensitivity of MRI.^3^ In the case series by Archarya et al, and in several cases reviewed for this report, extraocular muscle abscesses were well visualized on CT.^2^,^5^,^8^ In the pediatric case report by Haufschild et al, MRI was used.^6^ Given the rarity of muscular abscesses in this specific location, the best imaging modality has not been determined; however, both MRI and CT seem to be effective.

Traditional treatments have demonstrated the efficacy of IV antibiotics and surgical drainage of extraocular muscle abscesses.^1^ In this case report, IV antibiotics alone were insufficient in reducing the size of the abscess, suggesting that a combination of surgical intervention such as orbitotomy and IV antibiotics may be necessary for effective treatment. Unfortunately, although surgical drainage was recommended and even scheduled by ophthalmology during the inpatient stay, the patient chose to leave against medical advice, opting for oral antibiotics and outpatient follow up with ophthalmology for a procedure if indicated. Although the patient leaving against medical advice limited the ability to assess the efficacy of surgical intervention, the lack of improvement on IV antibiotics and the specialist’s recommendations for surgical drainage underlines that oral or intravenous antibiotic therapy may be insufficient as a standalone treatment. Furthermore, administration of steroids was described in one documented case but was not shown to be an effective treatment for reducing swelling or associated symptoms. 2 There is also a documented case where intravenous Co-amoxiclav was effective in treating an inferior rectus abscess caused by pyomyositis, eliminating the need for surgical interventions.^10^ This case – a successful improvement of symptoms with antibiotics – showcases the variability in treatment response. Therefore, the most effective approach may resemble that for many abscesses, involving the prescription of antibiotics (either intravenously or orally) in conjunction with orbitotomy for fluid drainage and infection control, along with methods to reduce swelling, pain, and other associated symptoms.

While these cases are rare, abscesses in the ocular regions may call for urgency. In a retrospective study of 31 patients with subperiosteal orbital abscesses (SPOAs), 30 patients underwent surgical drainage within two days of confirmed diagnosis. However, one patient who underwent surgical drainage on day three developed diplopia, highlighting the potential risks of delayed intervention as symptoms can progress with time.^11^ Furthermore, a significant morbidity persists in some cases of orbital abscess despite intravenous antibiotic therapy and surgical drainage. A review of 15 cases revealed complications such as visual loss (four patients), residual proptosis (two patients), residual diplopia (two patients), osteomyelitis (one patient), and death (one patient). Crucially, these outcomes occurred despite intravenous antibiotic therapy and surgical drainage, emphasizing the severe potential outcomes of this condition. It was noted that many of these patients received inappropriate or inadequate oral antibiotic therapy prior to referral, which likely contributed to their poor outcomes and emphasizes the importance of early recognition and appropriate management to minimize long-term severe complications.^12^

A more recent case reports the successful use of ultrasound-guided fine-needle aspiration for managing orbital abscesses on a patient with a subperiosteal orbital abscess with significant clinical improvement and no recurrence observed during a three-year follow-up. This innovative, minimally invasive technique suggests a promising additional option to traditional antibiotic therapy. Encouraging the integration of such minimally invasive approaches alongside medication could enhance treatment outcomes and expand treatment options for rare orbital abscess management. 13

As a single case study, the findings may not be generalizable to all patients with inferior rectus abscesses; therefore, additional studies may be needed to confirm the observations and recommendations. The patient’s decision to leave against medical advice limits the understanding of the long-term outcomes of the recommended treatment plan, particularly the effectiveness of surgical intervention.

This case report contributes to the expanding body of literature on this rare condition, emphasizing the critical importance of heightened awareness among emergency physicians. Despite its infrequent occurrence, this condition may result in impaired ocular motility and permanent vision loss. Therefore, it is important to include it in the differential diagnosis so that appropriate treatments, such as IV antibiotics and consultation with ophthalmology for possible drainage, can be initiated as quickly as possible.

## Supplementary Information












