# Supplementary figures and images for: A Case Report of Inferior Rectus Abscess
Source: J Educ Teach Emerg Med. 2025 Apr 30;10(2):V10–3. doi: 10.21980/J8J35G (PMC12054069; doi:10.21980/J8J35G)

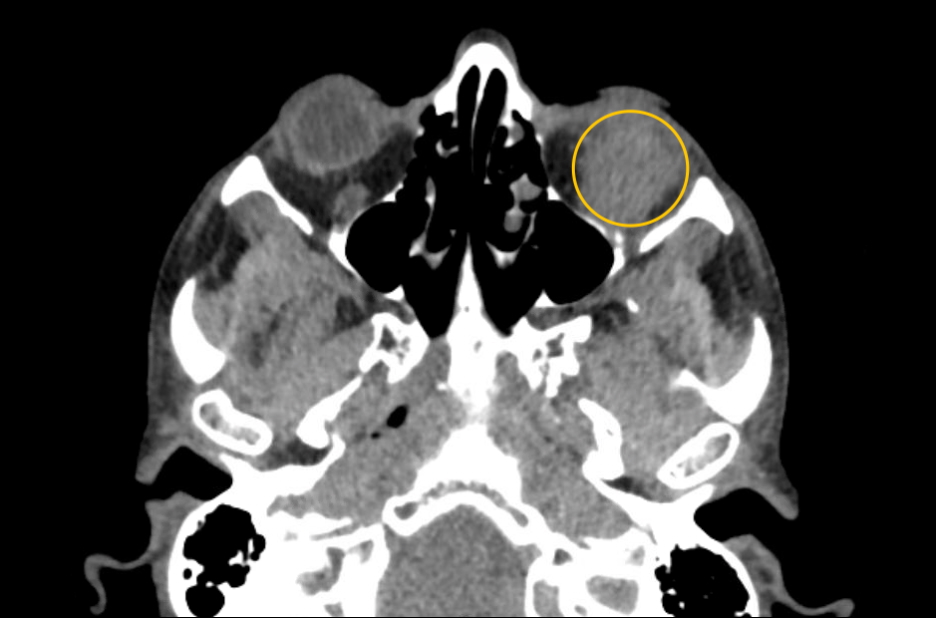

Supplement: Supplementary file 1 [file 10-2-V10-supp1.jpeg]

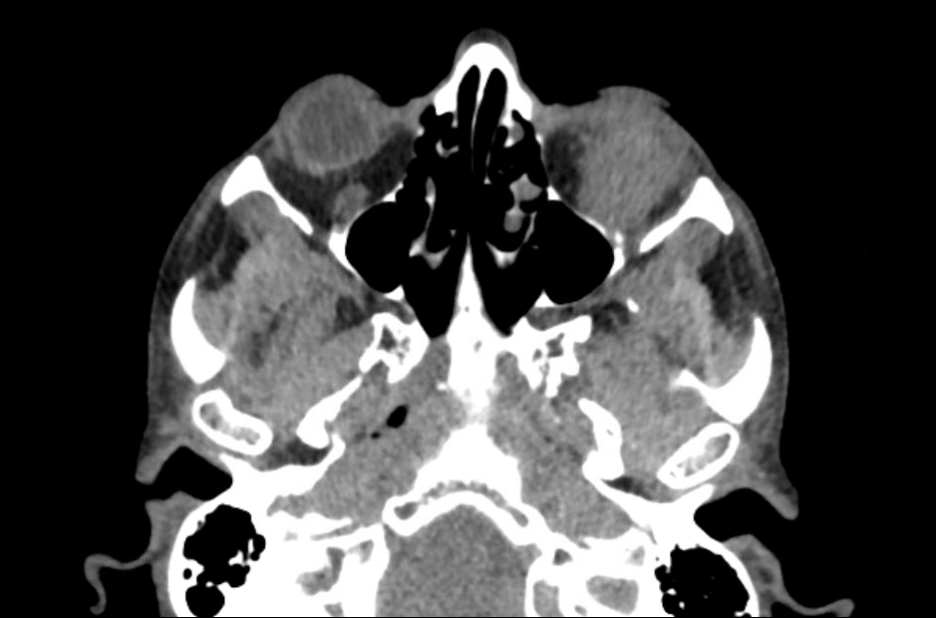

Supplement: Supplementary file 2 [file 10-2-V10-supp2.jpeg]

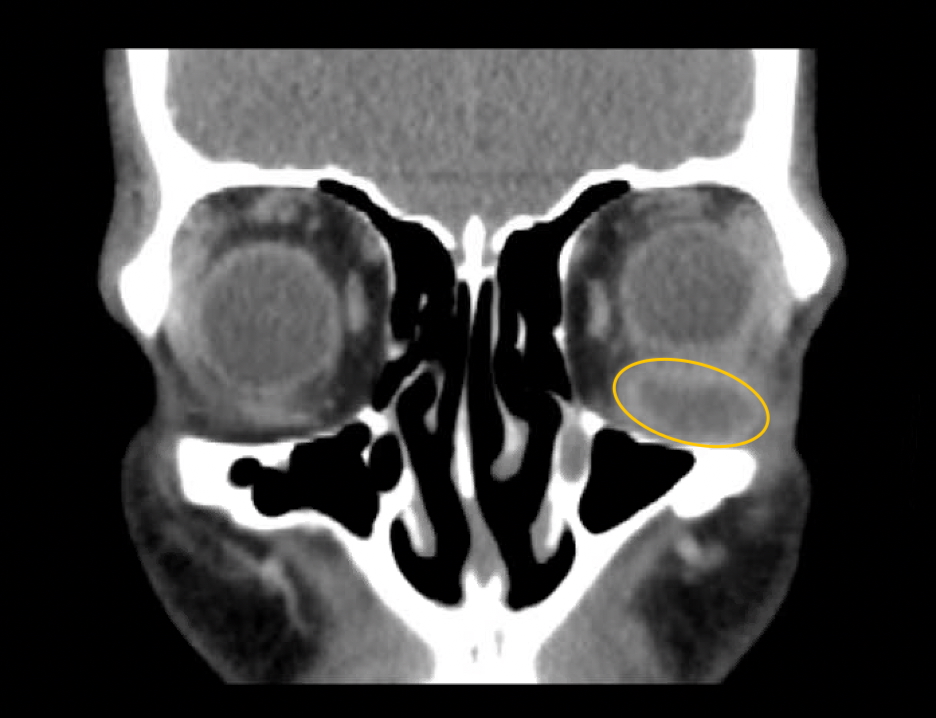

Supplement: Supplementary file 3 [file 10-2-V10-supp3.jpeg]

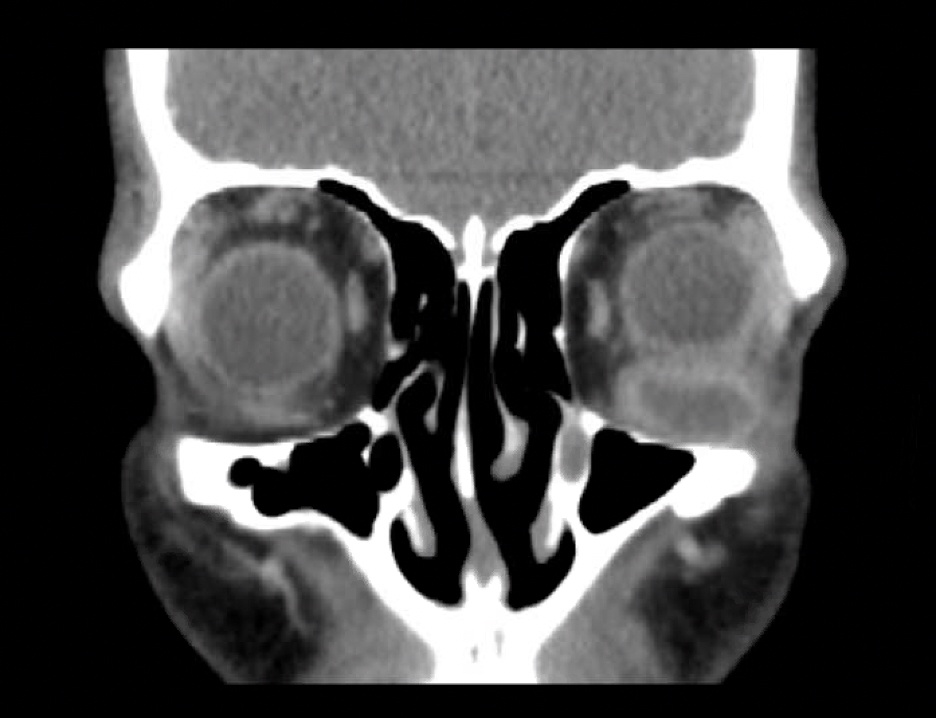

Supplement: Supplementary file 4 [file 10-2-V10-supp4.jpeg]

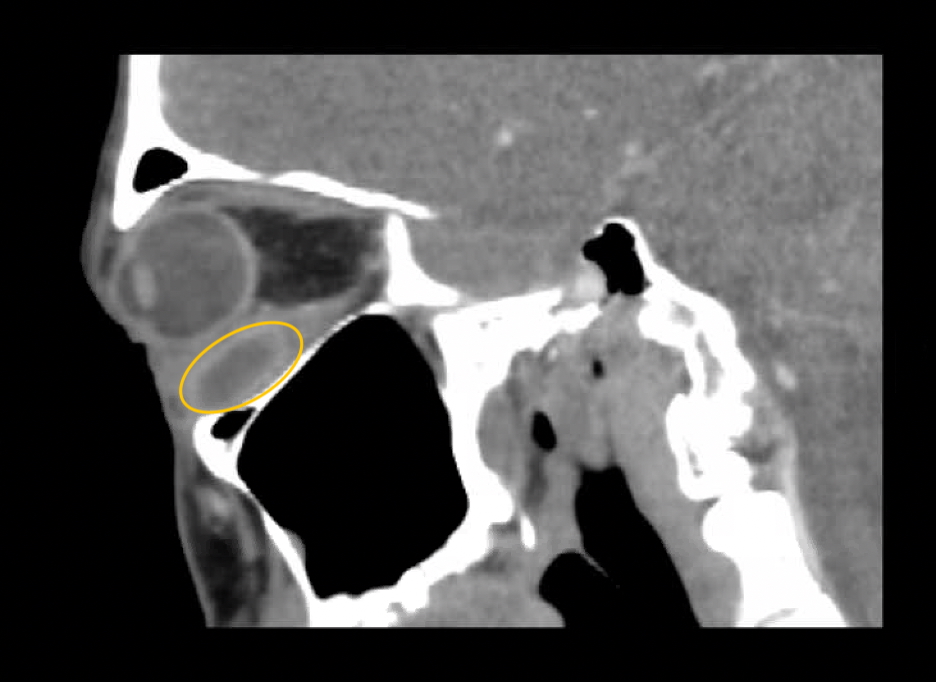

Supplement: Supplementary file 5 [file 10-2-V10-supp5.jpeg]

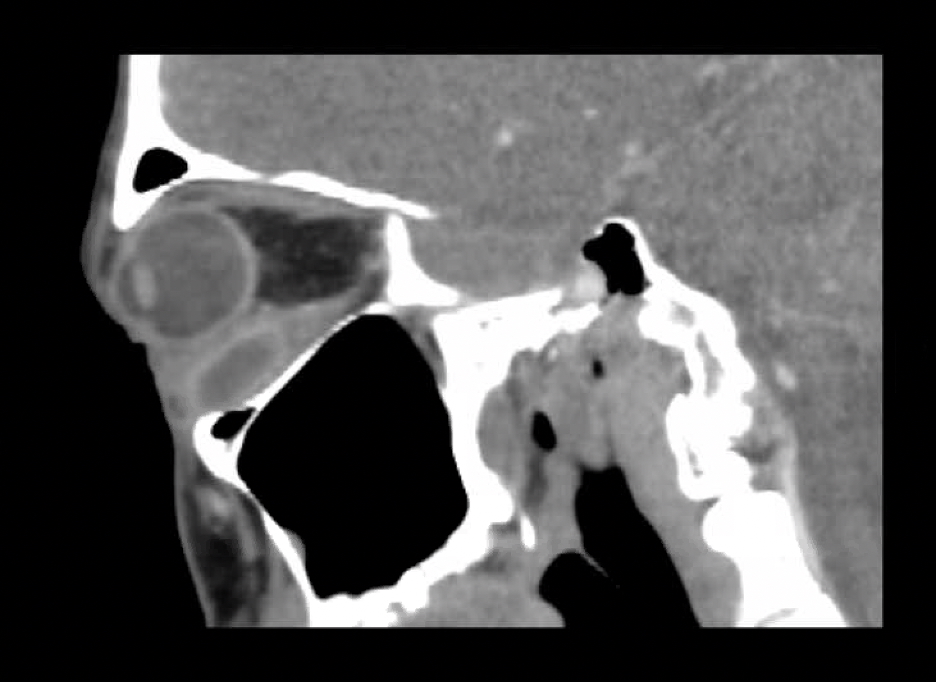

Supplement: Supplementary file 6 [file 10-2-V10-supp6.jpeg]
